# Iron overload in polytransfused patients without heart failure is associated with subclinical alterations of systolic left ventricular function using cardiovascular magnetic resonance tagging

**DOI:** 10.1186/1532-429X-13-23

**Published:** 2011-04-26

**Authors:** Stéphanie Seldrum, Sophie Pierard, Stéphane Moniotte, Christiane Vermeylen, David Vancraeynest, Agnès Pasquet, Jean-Louis Vanoverschelde, Bernhard L Gerber

**Affiliations:** 1 Pôle de Recherche Cardiovasculaire, Institut de Recherche Expérimentale et Clinique, Cliniques Universitaires St-Luc and Université Catholique de Louvain, Brussels Belgium

## Abstract

**Background:**

It remains incompletely understood whether patients with transfusion related cardiac iron overload without signs of heart failure exhibit already subclinical alterations of systolic left ventricular (LV) dysfunction. Therefore we performed a comprehensive evaluation of systolic and diastolic cardiac function in such patients using tagged and phase-contrast CMR.

**Methods:**

19 patients requiring regular blood transfusions for chronic anemia and 8 healthy volunteers were investigated using cine, tagged, and phase-contrast and T2* CMR. LV ejection fraction, peak filling rate, end-systolic global midventricular systolic Eulerian radial thickening and shortening strains as well as left ventricular rotation and twist, mitral E and A wave velocity, and tissue e' wave and E/e' wave velocity ratio, as well as isovolumic relaxation time and E wave deceleration time were computed and compared to cardiac T2*.

**Results:**

Patients without significant iron overload (T2* > 20 ms, n = 9) had similar parameters of systolic and diastolic function as normal controls, whereas patients with severe iron overload (T2* < 10 ms, n = 5), had significant reduction of LV ejection fraction (54 ± 2% vs. 62 ± 6% and 65 ± 6% respectively p < 0.05), of end-systolic radial thickening (+6 ± 4% vs. +11 ± 2 and +11 ± 4% respectively p < 0.05) and of rotational twist (1.6 ± 0.2 degrees vs. 3.0 ± 1.2 and 3.5 ± 0.7 degrees respectively, p < 0.05) than patients without iron overload (T2* > 20 ms) or normal controls. Patients with moderate iron overload (T2* 10-20 ms, n = 5), had preserved ejection fraction (59 ± 6%, p = NS vs. pts. with T2* > 20 ms and controls), but showed reduced maximal LV rotational twist (1.8 ± 0.4 degrees). The magnitude of reduction of LV twist (r = 0.64, p < 0.001), of LV ejection fraction (r = 0.44, p < 0.001), of peak radial thickening (r = 0.58, p < 0.001) and of systolic (r = 0.50, p < 0.05) and diastolic twist and untwist rate (r = -0.53, p < 0.001) in patients were directly correlated to the logarithm of cardiac T2*.

**Conclusion:**

Multiple transfused patients with normal ejection fraction and without heart failure have subclinical alterations of systolic and diastolic LV function in direct relation to the severity of cardiac iron overload. Among all parameters, left ventricular twist is affected earliest, and has the highest correlation to log (T2*), suggesting that this parameter might be used to follow systolic left ventricular function in patients with iron overload.

## Introduction

Repeated transfusion of packed red cells in patients with chronic anemia leads to chronic iron overload in many different organs, and especially the heart. Such chronic cardiac iron overload can cause heart failure, systolic [1-3] and diastolic dysfunction, arrhythmias and is a leading cause of mortality in such patients with multiple transfusions.

T2* measurement by cardiovascular magnetic resonance (CMR) is a novel technology which allows to non-invasively assess the severity of hepatic and cardiac iron overload [4-7]. In the liver, R2* the inverse of T2*, was shown to directly correlate with iron concentration by biopsy. In the heart, R2* was shown to be linearly correlated to cardiac iron content quantified in an animal model of iron overload in gerbils [8] and in a postmortem study of a thalassemia patient [9]. The monitoring of cardiac T2* has important clinical impact for treatment of patients with chronic anemia. Indeed, severe cardiac iron overload in patients with cardiac T2* lower than < 10 ms was shown to predict increased risk of development of heart failure and of sudden cardiac death [10]. Fortunately iron can be removed from the body by chelators, and T2* CMR [11-13] can be used to evaluate the efficacy of such chelation therapy [11-13], to reduce cardiac iron content. This was shown to improve ejection fraction [11-13], and to decrease mortality due to heart failure and sudden death [14]. Therefore such regular monitoring of cardiac iron overload by cardiac T2* is now recommended [15] for guiding and monitoring the efficacy of iron chelation therapy in chronically transfused patients.

Yet, the impact of moderate iron overload on alterations of left ventricular function still remains incompletely understood. Indeed an inverse curvilinear relation between T2* and left ventricular (LV) ejection fraction (EF) was demonstrated when T2* was smaller than 20 ms [4,16]. Yet, these studies included up to 20% of patients with overt heart failure and reduced ejection fraction. Therefore we sought to evaluate whether such patients with iron overload but without clinical signs of heart failure, and with apparently preserved LV ejection fraction, already present subclinical signs of systolic and diastolic dysfunction. Also we wanted to investigate whether more sophisticated parameters of systolic cardiac function, such as strains or rotational twist, would be affected earlier than LV ejection fraction in patients with moderate iron overload. Accordingly, in the current study we performed a comprehensive assessment of systolic and diastolic function by means of tagged and phase contrast CMR in 19 patients with anemia and 8 healthy volunteers, and we compared the magnitude of alteration of these parameters versus the magnitude of cardiac iron overload.

## Materials and methods

### Patients and Study Protocol

This prospective study included consecutive patients with a chronic anemia treated by transfusion of packed red cells, who underwent a clinically indicated T2* CMR study to quantify myocardial iron content. Only healthy patients without clinical signs of heart failure and with normal cardiac function by echocardiography were considered for inclusion into this study. Patients with signs or history of heart failure, depressed LV-function, significant valve disease, or cardiac arrhythmia were excluded. We thus studies 19 patients with anemia (Table 1) and compared them to 8 age matched healthy volunteers without a hematological disease undergoing the same CMR protocol. The study protocol was approved by the Institutional Review Board of our University and participants gave informed consent to participating in the study.

**Table 1:** Characteristics of the study population

	Patients (n = 19)	Controls (n = 8)	P value
Age	24 ± 8 (11-35)	30 ± 3 (26-37)	0.05
Sex	9M/10F	4M/3F	(NS)
Weight (kg)	48 ± 11	65 ± 11	<.001
Height (cm)	149 ± 13	175 ± 10	<.001
BSA (m^2^)	1.4 ± 0.2	1.8 ± 0.2	<.001
BP systolic (mmHg)	105 ± 13	107 ± 15	.96
BP diastolic (mmHg)	62 ± 7	60 ± 10	.42
Heart Rate (bpm)	78 ± 15	64 ± 7	.02
			
Hb value	9.2 ± 1.2	-	-
Ferritin	1686 ± 914	-	-
T2* cardiac (ms)	22 ± 11 (6-44)	40 ± 10 (24-59)	<.001
T2* hepatic (ms)	6 ± 7 (1-24)	21 ± 4 (15-26)	<.001

BP: Blood pressure. BSA: Body surface area. Hb: Hemoglobin

### MRI acquisition

Acquisitions were performed using a 1.5 Tesla magnet (Intera CV, Philips) with a 5 element phased array coil. T2* measurement of the liver was performed using single-breath-hold, 20-echo (1.07 to 21 ms) gradient echo sequence in an 10 mm axial slice through the liver. Repetition time (TR) was 150 ms, field of view 360 mm, image matrix 176 × 176 and flip angle 35°. Then, after horizontal and long-axis localizers, myocardial T2* was assessed from a midpapillary ventricular short-axis slice using a cardiac-gated, segmented, multiecho gradient echo sequence obtained in a single breath-hold, similar to the technique described by Westwood et al. [17]. Eight echoes with a minimum echo time (TE) of 2.0 ms, an echo spacing of 2.2 ms, and a repetition time of 19.1 ms were obtained. Next, for assessment of cardiac function, 8-10 contiguous short axis cine images covering the left-ventricle from base to apex, and 2, 3 and 4 chamber long axis cine-images were acquired using a retrospective ECG-gated segmented k-space steady state free precession pulse sequence (SSFP) (slice thickness 8 mm, spacing 2 mm, TR: 3 ms, TE: 1.5 ms, flip angle: 60°, 20 phases/view). Thereafter 8-10 contiguous tagged short-axis and 2- 3- 4 chamber long axis cine images of the left ventricle were acquired in the same directions using a prospective ECG-gated, segmented k-space gradient echo pulse sequence with echo-planar readout and spatial modulation of magnetization (SPAMM) applied in a 6 mm grid pattern. Imaging parameters were: slice thickness: 8 mm, spacing: 2 mm, TR: 12 ms, TE: 2.3 ms, image matrix: 256 × 160, field of view: 36 cm, flip angle: 12°, echo train length: 7-9. All images were acquired during short (10-15 seconds) breath holds in end-expiration.

Finally, mitral inflow velocity and mitral annular motion velocity were evaluated using a free breathing, ECG gated segmented K space gradient echo sequence prescribed in a short-axis orientation at the level of the mitral valve as described by Paelinck et al. [18]. Images were acquired with 63 phases encoded per heart beat resulting in an average temporal resolution of 13 ms (range 8-16 ms). Other imaging parameters were: TR 12 ms, TE 2.6 ms, matrix 224 × 178 pixels, flip angle 15°. Mitral inflow velocity imaging was encoded with a velocity of 250 cm/s. Mitral annular motion was encoded with a velocity of 30 cm/s.

### Computation of cardiac and hepatic T2* and R2*

Regions of interest were placed in the septal wall of the heart and in the liver. Cardiac and hepatic T2* times were computed by mono-exponential fit to the equation y = K e ^-TE/T2^*. R2* was computed as the reciprocal of T2*.

### LV volumes, Ejection fraction and PLVFR

Cine SSFP CMR images were analyzed by a blinded observer using the freely automated software SEGMENT [19]. The endocardium and epicardium of the left ventricle were fully automatically contoured on all phases of the left ventricle, with manual adjustments when needed. Left ventricular end-diastolic (LV-EDV) and endsystolic volumes (LV-ESV) were calculated using Simpson's method. The first image of the cardiac cycle was considered to be end-diastole, whereas the smallest volume of the LV curve was considered end-systolic volume. Peak LV filling rate (PLVFR) was computed as maximum of derivate of LV volume curve in diastole over time. LV mass was computed assuming a myocardial density of 1.06 and excluding papillary muscles. LV volumes and mass were indexed to body surface area. LVEF was computed as EDV-ESV/EDV.

Left atrial volume was computed at the time of maximal atrial filling just before mitral valve opening using the area-length method as  where A_LA2c _and A_LA4c _are the areas of the left atrium planimetered in 2 and 4 chamber views and l_LA _the length of the atrium respectively.

#### CMR Strain

**Figure 1:**
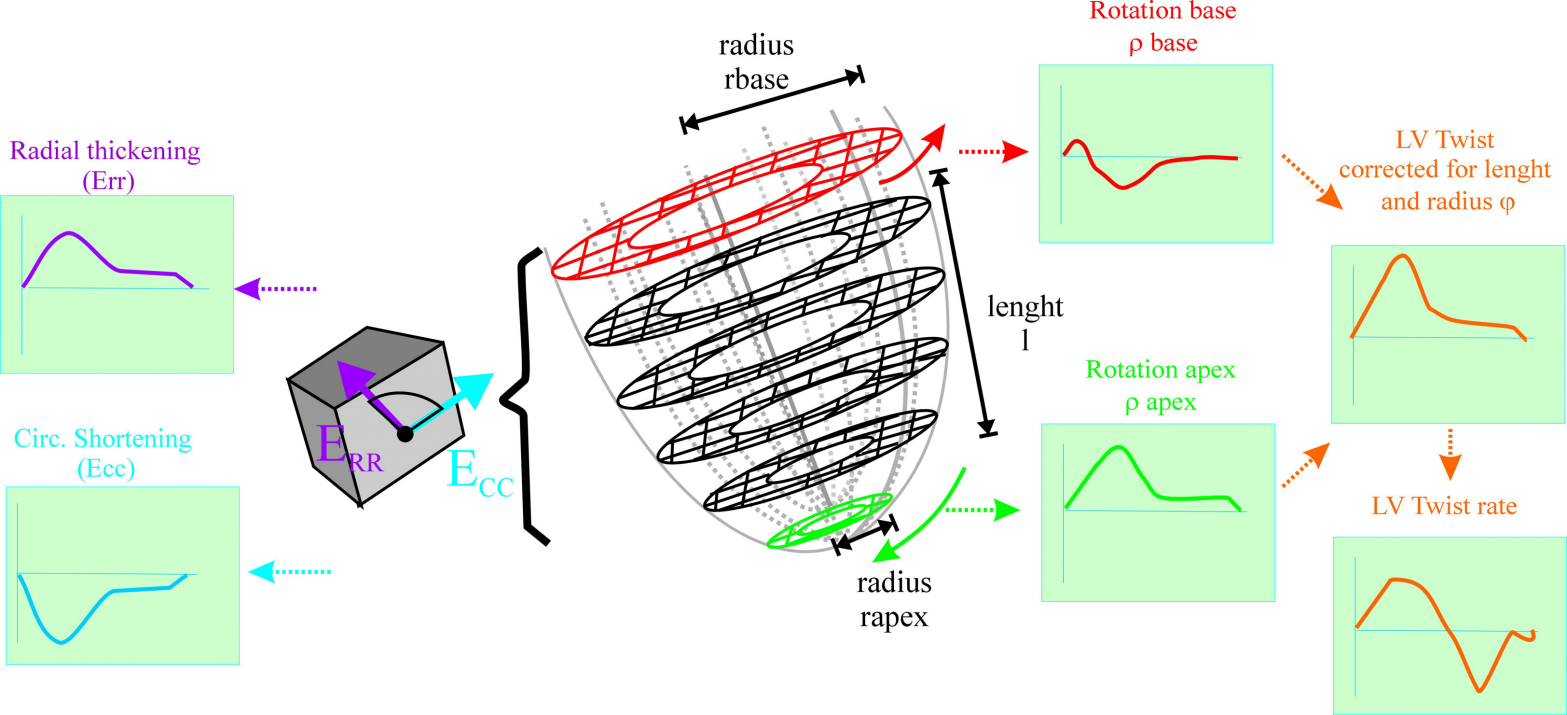
**Illustration of the measurements obtained from tagged MR**. Consecutive tagged short-axis planes were acquired from apex to base. On each short-axis plane, radial thickening (Err, purple) and circumferential shortening strain (Ecc, blue) were computed and the average of Err and Ecc for the entire left ventricle was recorded. Rotation of the most basal (red) and apical slice (green) slices were computed and left ventricular torsion (orange) was calculated as difference between apical and basal rotation divided by the length (l) between apex and base and multiplied by the mean radius of the base (r_base_) and apex (r_apex_) to obtain LV twist (φ). The first derivate of LV twist over time was respectively systolic twist rate and diastolic untwist rate.

Tagged CMR images were analyzed quantitatively using Harmonic Phase Imaging Analysis (HARP - Diagnosoft, CA) as previously described [20] and as illustrated in Figure 1. End-systolic systolic Eulerian circumferential shortening strain (Ecc) and radial thickening strain (Err) as well as circumferential-radial rotation were computed at the midwall level of each slice. The average of all slices per patients was used as global radial thickening, circumferential shortening strain and rotation. By convention, strains were defined to have a negative sign for shortening (active contraction) and a positive sign for elongation (passive deformation). LV torsion was computed as difference in rotation between the most apical and basal slices. LV twist (φ) was computed as torsion corrected for length: i.e.  where ρ is the rotation at the most basal and most apical slice respectively, l is the apex to base length and r is the LV radius at apex and base respectively. Strains were computed in end-systole, determined by the time of aortic valve closure on cine images.

The first derivative of twist versus time was computed to yield respectively systolic twist rate and diastolic untwist rate.

### Analysis of phase contrast imaging

**Figure 2:**
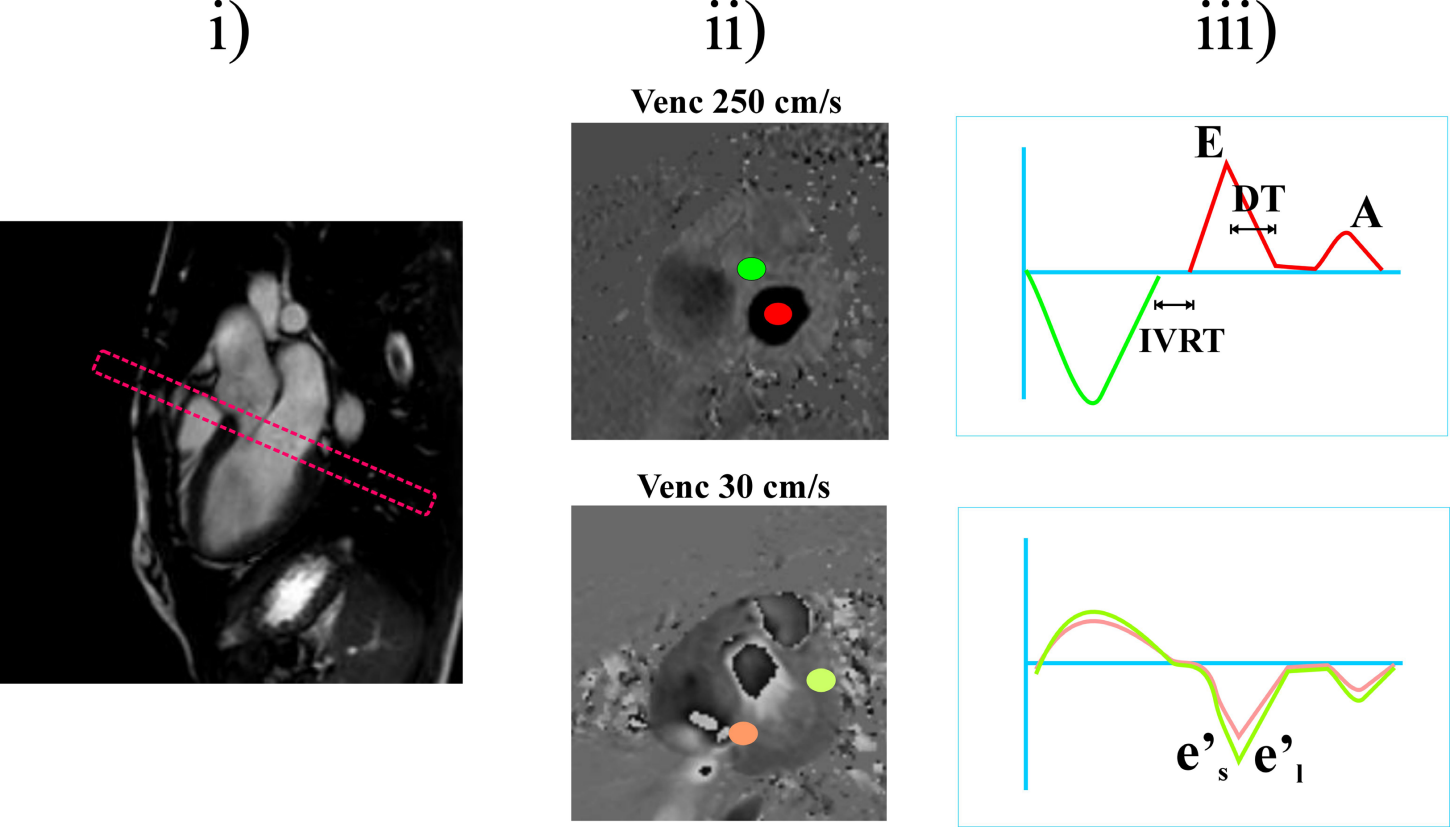
**Illustration of the prescription and measurements obtained from phase contrast MR**. Two identical stacks of phase contrast images were prescribed on a 3 chamber view of the heart (i). To assess transmitral and aortic flow, a velocity encoding (venc) of 250 cm/s was used and the center of the slice was positioned perpendicular to mitral inflow, at early diastole (ii, upper panel). To assess tissue MR velocities, phase-contrast MR was repeated with a velocity encoding of 30 cm/s, (ii, lower panel) To derive aortic and trans-mitral flow (iii, top panel), circular regions of interest were placed in the aortic (green) and mitral valve (red). On the corresponding mitral flow curve (panel iii, red) the peak mitral velocity of rapid early (E) filling wave late atrial (A) filling wave were recorded. The deceleration time (DT) of the early (E) wave of the mitral valve was computed between the peak of the E wave and the point where the fitted line of descending slope of the E wave reached zero velocity. The isovolumic relaxation time (IVRT) was computed as the delay between the end of the aortic valve flow and the beginning of the transmitral flow. On the tissue velocity images (ii lower panel, regions of interest were placed on the septal (orange) and lateral wall (yellow) and corresponding tissue velocity versus time curves were plotted (iii lower panel). From these curves, peak tissue velocity in early diastole in the septum (e'_s_) and lateral wall (e'_l_) was measured and average E/e'ratio was computed.

Phase contrast images were analyzed on a dedicated work station (Philips Medical Viewforum release 4.1). A region of interest was placed in the center of the mitral valve and in the aortic valve outflow tract and mean velocity of both regions was plotted over time (Figure 2). LV ejection time (LVET) was computed based on duration of aortic ejection. Isovolumic relaxation time (IVRT) was measured between the end of aortic ejection and the start of mitral filling. Peak early (E) inflow and late atrial (A) velocity were recorded and the E/A ratio were computed. The descending slope of the E wave was plotted and the deceleration time of the E wave (DT) was measured between the peak of the E wave and the point where the fitted line of descending slope of the E wave reached 0 velocity. Peak septal and lateral tissue annular velocity e's and e'l were computed in regions of interest placed in septum and lateral wall on images encoded with a velocity of 30 cm/s. Ratios of mitral E wave to tissue e's and e'l wave velocity were computed as described by Paelinck et al [18].

### Statistical analysis

All continuous values are reported as mean ± one SD. Patients with anemia were separated into 3 groups according to cardiac T2* times (< 10 ms, representative of severe iron overload, 11-20 ms, representative of moderate iron overload and > 20 ms representative of no iron overload). Mean values of LV dimensions, and various systolic and diastolic parameters by CMR were compared among patients with different cardiac T2* values and controls using one-way ANOVA. Individual comparisons were performed post-hoc using Bonferroni test. The various CMR parameters were also compared with cardiac and hepatic T2* and R2* measurement using Pearson's regression analysis. All tests were two sided and a P value < 0.05 was considered indicative of statistical significance.

## Results

### Study population

The clinical characteristics of the nineteen patients with anemia and normal controls are shown in Table 1. Fifteen patients had thalassemia major. One of them also had an associated sickle-cell mutation. Four patients had refractory anemia (respectively 2 Blackfan-Diamond, and 2 sideroblastic anemias). Patients were transfused an average of 3.8 ± 2.0 units of packed red cells per month. All patients had significantly increased plasma ferritin values and received iron chelation therapy: Seven patients were treated with SC deferoxamine monotherapy, 10 with oral deferasirox monotherapy and two were treated with an association of 2 chelators (respectively one patient with an association of oral deferiprine and SC deferoxamine; and another patient with oral deferiprine and deferasirox). Controls had no family history of hemochromatosis. No patient or control was under medication with cardiac activity.

Controls had significantly larger body size than patients. Also heart rate was significantly higher in anemia patients than in controls.

### T2* values

As expected, patients had significantly higher hepatic and cardiac T2* values than controls. There was considerable variation of cardiac T2* values in chronically transfused patients. In 9 patients T2* was normal (cardiac T2* > 20 ms), 5 others had moderate iron overload (cardiac T2* 11-20 ms) and 5 patients showed severe iron overload (cardiac T2* < 10 ms).

### Systolic and diastolic function in anemic patients according to magnitude of iron overload and vs. controls

All tagging studies were successfully completed and could be evaluated, however data from 4 phase contrast studies was missing or non evaluable. Results are shown in table 2. Because of their smaller body size, absolute LV dimensions, volumes and mass of patients with hematological disease were significantly smaller than those of controls. However, when adjusted to body surface, LV and atrial volumes and LV mass were not significantly different among patients and controls.

**Table 2:** Size and parameters of systolic and diastolic function and CMR in hearts of patients with multiple transfusion vs Controls

		patients				
		T2* ≤ 10 ms (n = 5)	T2* 11-20 ms (n = 5)	T2* > 20 (n = 9)	Controls (n = 8)	P value ANOVA
	LV mass (g/m2)	78 ± 21	65 ± 12	76 ± 17	68 ± 9	.21 (NS)
	Apex-Base lenght (mm)	63 ± 5¶	67 ± 5¶	60 ± 10¶	81 ± 6	<.001
	LV diameter base (mm)	41 ± 3	42 ± 5	48 ± 7	50 ± 6	.02
**LV Size**	LV EDVi (ml/m2)	95 ± 16	81 ± 18	83 ± 14	84 ± 7	.63 NS)
	LV ESVi (ml/m2)	44 ± 9	35 ± 13	32 ± 9	29 ± 6	.11 (NS)
	LA area (ml/m2)	46 ± 11	39 ± 12	35 ± 10	43 ± 16	.42 (NS)


	LV EF (%)	54 ± 3¶	59 ± 7	62 ± 6	65 ± 6	.01
	Ejection time (ms)	267 ± 20	280 ± 27	263 ± 35¶	306 ± 13	.06 (NS)
	LV Rotation apex (°)	7 ± 2¶	9 ± 4¶	11 ± 5¶	19 ± 6	<.001
**Systolic parameters**	LV Rotation base (°)	-2 ± 1	-3 ± 2	-4 ± 3	-3 ± 2	.44 (NS)
	LV Torsion uncorrected(°)	10 ± 2¶	12 ± 4¶	15 ± 6¶	23 ± 5	<.001
	LV Twist **φ **corrected for LV length and radius (°)	1.6 ± 0.2¶†	1.9 ± 0.5¶	3.0 ± 1.2	3.5 ± 0.7	.002
	End-systolic Ecc (%)	-10 ± 3	-14 ± 3	-12 ± 4	-16 ± 3	.05
	End-systolic Err (%)	+6 ± 4¶	+9 ± 2	+11 ± 1	+11 ± 4	.05
	Peak systolic twist rate (°/msec)	7 ± 2	9 ± 3	12 ± 5	13 ± 4	.07 (NS)

	PFR (ms)	581 ± 188	504 ± 232	643 ± 191	700 ± 134	.27 (NS)
	E wave amplitude (cm/s)	77 ± 3	78 ± 12	69 ± 9	64 ± 10	.08 (NS)
	A wave amplitude (cm/s)	29 ± 3	34 ± 12	36 ± 6	37 ± 12	.66 (NS)
**Diastolic parameters**	E/A ratio	2.7 ± 0.2	2.6 ± 1.1	2.0 ± 0.4	2.2 ± 0.8	.61 (NS)
	DT (ms)	131 ± 16	116 ± 20	128 ± 29	154 ± 31	.21(NS)
	E' wave amplitude septum (cm/s)	13 ± 8	10 ± 3	11 ± 5	11 ± 3	.91 (NS)
	E' wave amplitude lateral wall (cm/s)	19 ± 7	13 ± 5	17 ± 5	15 ± 2	.67 (NS)
	E/e' septum	8 ± 5	8 ± 3	7 ± 2	6 ± 2	.50 (NS)
	E/e' lateral	4 ± 1	7 ± 4	4 ± 2	4 ± 1	.25 (NS)
	IVRT (ms)	50 ± 4	71 ± 19	65 ± 10	73 ± 20	.48 (NS)
	Peak diastolic untwist rate (°/msec)	-7 ± 2	-9 ± 2	-18 ± 11	-17 ± 10	.10 (NS)

LV: Left ventricular, EDVi: indexed End-diastolic volume, ESVi indexed End-systolic volume, LA: Left atrial, EF: Ejection fraction, Ecc: Eulerian circumferential shortening, Err: Eulerian radial Thickening, PFR Peak filling rate, DT: Mitral E wave deceleration time, IVRT: Isovolumic relaxation time.

¶ p < 0.05 vs controls. † p < 0.05 vs T2* > 20 ms by Bonferroni test

By study design, all patients had LV ejection fraction > 50%. In patients without significant iron overload, LV ejection fraction and strains were not significantly different from controls. Rotation of the LV base was similar in patients and in controls. However the rotation of the apex and uncorrected LV torsion were significantly lower in patients than in controls. This was due to shorter LV length and diameter of the hearts of patients. Indeed, when corrected for LV length and diameter, LV twist was similar in patients without significant iron overload (T2* > 20 ms) and in controls.

Asymptomatic patients with severe cardiac iron overload (cardiac T2* < 10 ms) displayed significant reductions of LV ejection fraction, radial thickening and LV torsion and LV twist as compared to controls and patients with T2* > 20 ms. In patients with moderate cardiac iron overload (cardiac T2* 10-20 ms), LV ejection fraction was preserved, however LV twist was significantly reduced as compared to controls.

Among all diastolic parameters studied, none did attain statistical significance.

### Correlations of parameters versus cardiac and hepatic T2* and R2* values

**Figure 3:**
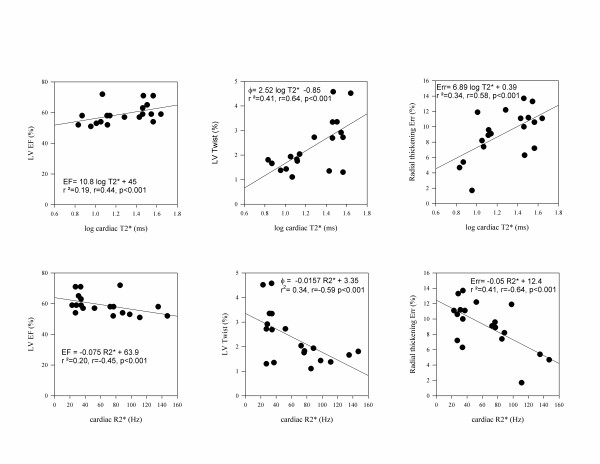
Correlation of parameters of systolic function vs. cardiac log T2* and R2* in patients with multiple transfusion.

The magnitude of reduction of LVEF, of systolic radial thickening and systolic LV twist and systolic LV twist rate in patients correlated linearly with the severity of cardiac but not with hepatic iron overload (Table 3, Figure 3). Among diastolic parameters, only rotational untwist rate correlated inversely and significantly both with the severity of cardiac and hepatic iron overload.

**Table 3:** Significant correlations of Systolic and Diastolic Parameters with cardiac and hepatic T2* and R2* in patients with chronic transfusion

	r vs.			
Parameter	Log cardiac T2*	Cardiac R2	Log hepatic T2*	Hepatic R2*
LV twist φ	**0.64¶**	**-0.59¶**	0.09	-0.17
LV EF	**0.44¶**	**-0.45¶**	0.33	-0.36
End-systolic Err	**0.58¶**	**-0.64¶**	0.09	-0.18
Peak systolic twist rate	**0.50¶**	**-0.49¶**	0.10	-0.21
Peak diastolic untwist rate	**-0.53¶**	**0.49¶**	**-0.50¶**	0.39

LV: Left ventricular, EF: Ejection fraction, Err: Eulerian radial Thickening

¶p < 0.05

## Discussion

This study evaluated systolic and diastolic function by tagged and phase contrast CMR in patients with repeated transfusion. The originality of our study is twofold: Indeed as opposed to earlier work, we studied only asymptomatic patients without clinical signs of heart failure and with preserved ejection fraction. In addition, we computed myocardial strains from tagged MR to precisely evaluate myocardial contractility in our patients.

### Alterations of systolic and diastolic function in iron overload

Earlier work demonstrated that LV ejection fraction is reduced in patients with severe cardiac iron overload [1-4,16]. Other studies [21-27] employing tissue Doppler echocardiography, described reductions of systolic tissue Doppler velocities in patients with chronic iron overload. Hamdy et al [25] also reported reduced systolic strains using tissue Doppler echocardiography in patients with iron overload. Our study is the first to evaluate myocardial strains by tagged CMR in patients with iron overload.

Our study corroborates earlier reports of reduced systolic function in severe cardiac iron overload [1-4,16]. Indeed, our study demonstrates significant reductions of LV ejection fraction and of LV strains in hearts with severe iron overload (T2* < 10 ms). Furthermore, we were also able to demonstrate that hearts with moderate iron overload (T2* 10-20 ms) already presented significant reductions of LV rotation twist, even when LV ejection fraction was still maintained. We also observed that the magnitude of alterations of all parameters of systolic function correlated linearly with the severity of iron overload measured either as log T2* or R2*. Thereby we confirmed the previously reported inverse relation between LV ejection fraction and T2*, but extended this observation to patients with overall preserved ejection fraction and without heart failure. Of all parameters studied, we observed that LV rotational twist was affected earliest, and had the highest correlation with the severity of cardiac iron overload. Our study thus stresses the value of LV strains and in particular of LV torsion and twist, as early markers of LV systolic dysfunction in patients with cardiac iron overload. This finding is consistent with other reports, which demonstrated that LV rotation and torsion are highly sensitive indicators of early LV systolic dysfunction in ischemic heart disease, pressure overload, cardiomyopathy and diabetes mellitus [28-30].

In contrast to systolic function, our study found no alterations in diastolic function in patients with iron overload. The only parameter which correlated significantly with the severity of iron overload in patients was diastolic LV untwisting velocity. Also in the literature, reports on diastolic dysfunction in patients with iron overload are conflicting. Indeed some studies using echocardiography [21,22,24,31,32], reported alterations of E/A ratio and E/e'ratios in multiple transfused patients when iron overload by T2* was present. On the other hand, Leonardi et al. [16] reported, similar to our findings, poor correlation between various diastolic parameters by echocardiography and T2* measurement by CMR in patients with iron overload. Also, Westwood et al. [33] reported poor correlation between T2* measurement and early and late diastolic filling rates by MR.

Interestingly in the present study we did not observe increases in atrial volumes, or in LV mass in patients with iron overload, as reported by others [4,22,34]. A potential explanation for these discrepancies might be that our present study excluded patients with heart failure symptoms and severely depressed cardiac function.

### Clinical Implications

The findings of the present study illustrate the harmful effects of iron overload on systolic function even before overt cardiac dysfunction or heart failure develops. Conventional echocardiographic techniques have failed to distinguish LV function of patients with thalassemia and iron overload from that of normal controls when global function was examined. Our study suggests that myocardial strains and in particular LV torsion and twist are more sensitive parameters than global LV ejection fraction for early detection of systolic dysfunction in patients with iron overload. Also LV untwisting velocity was the only useful parameter of diastolic dysfunction correlating with iron overload. This is particularly interesting, since speckle tracking echocardiography can now evaluate strains and in particular LV torsion and twist non-invasively. In principle, this technique might thus also be useful for following patients with chronic iron overload. Unfortunately, as we have previously shown [35], 2D echocardiography is limited by the inability to reliably visualize the true LV apex from parasternal views. Therefore its reproducibility is poor for evaluation of LV torsion and it may underestimate LV torsion and twist as opposed to CMR. Recently introduced 3D speckle tracking techniques [36] can visualize the entire heart including the true apex and thus promise more accurate and reproducible quantification of left ventricular rotation and twist. This might allow echocardiographic assessment of LV twist in patients with iron overload.

### Limitations

Severe iron overload causes magnetic susceptibility effects which may affect to some extent the image quality of our tagging sequence with echo-planar readout. Although the sharpness and persistence of tags were found to be reduced in some patients with severe iron overload in late diastole, we could compute strain in systole and early diastole in all patients. Another limitation is that we assessed diastolic function using a phase contrast imaging, with a lower temporal resolution compared to Doppler echocardiography. This may have affected our ability to precisely evaluate parameters of diastolic dysfunction. Yet this approach was shown in a validation study to provide as accurate measurements of diastolic function as Doppler echocardiography does [18].

## Conclusions

We have shown in a small patient cohort that LV rotational twist is the earliest and most affected parameter in hearts with moderate iron overload, and shows the best correlation versus the severity of cardiac iron overload. This suggests that this parameter might be useful to monitor development of LV systolic dysfunction in patients with cardiac iron overload.

## Competing interests

The authors declare that they have no competing interests.

## Authors' contributions

SS, SP, SM and BG: acquisition of CMR studies. SM and CV: patient recruitment. SS and BG: study design, analysis of data and manuscript redaction. AP, JLVO and DV study design and review of manuscript. All authors read and approved the final manuscript.

## Funding Source

Grant support by the Fondation Nationale de la Recherche Scientifique of the Belgian Government (FRSM 3.4557.02)
